# C2orf40 inhibits metastasis and regulates chemo-resistance and radio-resistance of nasopharyngeal carcinoma cells by influencing cell cycle and activating the PI3K/AKT/mTOR signaling pathway

**DOI:** 10.1186/s12967-022-03446-z

**Published:** 2022-06-08

**Authors:** Zuozhong Xie, Wei Li, Jingang Ai, Jun Xie, Xiaowei Zhang

**Affiliations:** 1 Department of Otolaryngology Head and Neck Surgery, The Third Xiangya Hospital, Central South University, Changsha, 410013 Hunan China; 2 Xiangya Clinical Research Center for Cancer Immunotherapy, Xiangya Hospital, Central South University, Changsha, 410008 Hunan China; 3 Department of Otolaryngology Head and Neck Surgery, Hunan Children’Hospital, Changsha, 410007 Hunan China

**Keywords:** Nasopharyngeal carcinoma, Metastasis, Prognosis, C2orf40, PI3K/AKT/mTOR signaling pathway

## Abstract

**Background:**

Nasopharyngeal carcinoma (NPC) is a malignant tumor of epithelial origin in head and neck with high incidence rate in Southern China. C2orf40 has been identified as a tumor suppressor gene in many cancers. However, the roles of C2orf40 in nasopharyngeal carcinoma has not been studied.

**Methods:**

In this study, a bioinformatics analysis was performed to identify the differentially expressed genes in NPC. The quantitative methylation levels was detected using pyrosequencing. qRT-PCR, western blotting, immunohistochemistry and immunofluorescence were used to detect the expression level of related RNA and proteins. Cell proliferation was detected using CCK-8 assay, and colony formation capability was detected using colony formation assays. Cell migration and invasion were analyzed using wound-healing and Transwell assays, respectively. The apoptosis level of cells was assessed using TUNEL staining. Endogenous DNA damage and repair were assessed by the comet assay. Cell cycle analyses carried out by flow cytometry. Finally, We used a xenograft nude mouse to verify the roles of C2orf40 in chemoresistance and radioresistance in vivo.

**Results:**

We found that the C2orf40 expression was significantly downregulated in NPC tissues and inversely associated with a poor prognosis. In vivo and in vitro functional experiments confirmed that overexpression of C2orf40 significantly inhibited the migration and invasion of NPC cells, and promoted their sensitivity to radiotherapy and chemotherapy of NPC cells. Mechanically, the expression level of C2orf40 was negatively correlated with the expression levels of CCNE1 and CDK1. Overexpression of C2orf40 induced cell cycle arrest of NPC cells at G/M phase. In addition, C2orf40 can down-regulated the expression levels of homologous recombination-related proteins (BRCA1, BRCA2, RAD51, and CDC25A) and inhibited the activity of the PI3K/AKT/mTOR signaling pathway.

**Conclusion:**

The results clarified the biological functions and mechanisms of C2orf40, as a tumor suppressor gene, in NPC, and provided a potential molecular target for improving the sensitivity of NPC to radiotherapy and chemotherapy.

## Introduction

Nasopharyngeal carcinoma (NPC) is a rare malignancy worldwide, while it is particularly common in the Southern China [1, 2]. More than 70% of patients with NPC are initially diagnosed with a locally advanced cancer because of concealed symptoms and invasiveness [3]. To date, chemo-radiotherapy has become the main treatment for NPC, owing to its high radio-resistance and chemo-sensitivity [4]. Although the combination of radiotherapy and chemotherapy has achieved a satisfactory 5 year survival rate (85–90%), 8–10% of patients had recurrence and tumor metastasis [5, 6]. However, the mechanisms underlying radio-resistance and chemo-resistance of NPC have still remained elusive [7, 8]. Thus, it is urgent to explore new treatment strategies to reveal these mechanisms for NPC.

In the process of comprehensive analysis of data collected from Gene Expression Omnibus (GEO) database, we found that the mRNA expression level of C2orf40 gene was significantly reduced in NPC cells. In China, C2orf40, also known as esophageal cancer-related gene-4 protein (ECRG4), was preliminarily identified and cloned by comparing the differential gene expression in normal esophageal epithelial cells and in esophageal squamous cell carcinoma (ESCC) cells [9]. In human, constitutive expression of Ecrg4 in a quiescent state monitors tissue homeostasis. Ecrg4 encodes a non-canonical hydrophobic leader sequence that assists in tethering Ecrg4 to the cell surface [10]. Upon injury, Ecrg4 is shed immediately from the surface and its gene expression rapidly decreases within 24 h, which is accompanied by the activation of inflammatory cascades [11]. Besides, naïve T cells highly express Ecrg4, which is down-regulated upon activation [12]. Although C2orf40 is highly expressed in various normal tissues, its expression is down-regulated in diverse types of cancer, including esophageal cancer [13], breast cancer [14], anaplastic thyroid carcinoma [15], and liver cancer [16], which may be partially due to the DNA hypermethylation at the promoter region [17, 18]. Especially, C2orf40 is a tumor suppressor gene and an independent prognostic factor of ESCC [19]. However, the mechanism of down-regulation of C2orf40 expression and its biological role in NPC have not been fully explored.

In the current study, based on the analysis of three GEO datasets (GSE12452, GSE53819, and GSE12452), we compared the gene expression profile data of normal nasopharyngeal epithelial tissues and NPC tissues, and it was found that the C2orf40 expression in NPC tissues was significantly down-regulated. Survival analysis indicated that decreased expression of C2orf40 was correlated with a poor prognosis of patients with NPC. In vitro and in vivo functional experiments revealed that C2orf40 not only promoted the sensitivity of NPC cells to chemotherapy and radiotherapy by inducing cell cycle arrest at G2/M phase and cell apoptosis, but also inhibited the migration ability of NPC cells.

## Materials and methods

### Cell culture

The human immortalized nasopharyngeal epithelial cell line (TERT) was cultured in a keratinocyte/serum-free medium supplemented with growth factors (Gibco Inc., Grand Island, NY, USA). Seven human NPC cell lines, including CNE-1, CNE-2, HONE-1, SUNE-1, HNE-1, HK-1, and 5-8F, were grown in a Roswell Park Memorial Institute (RPMI)-1640 medium (Corning Inc., Corning, NY, USA) supplemented with 10% fetal bovine serum (FBS) (Gibco, Grand Island, USA) in the presence of 5% CO_2_ at 37 °C. All NPC cell lines were purchased from the Cell Bank of the Type Culture Collection of Chinese Academy of Sciences (Shanghai, China). All cell lines were routinely tested for mycoplasma contamination and found to be negative.

### Data collection

The GEO database was used to obtain the gene expression profiles of human normal nasopharyngeal epithelial tissues and NPC tissues. The gene expression profiles and clinical data of GSE12452 (31 NPC samples and 10 controls), GSE53819 (18 NPC samples and 18 controls), and GSE64634 (12 NPC samples and 4 controls) datasets were downloaded.

### Collection of human tissue specimens

In the present study, human NPC tissues (*n* = 20) and human normal nasopharyngeal epithelial tissues (*n* = 12) were collected from The Third Xiangya Hospital of Central South University according to the ethical and legal standards of The Third Xiangya Hospital of Central South University. The diagnosis of primary NPC was confirmed by hematoxylin–eosin (H&E) staining by experienced pathologists. Written informed consent was obtained from all patients.

### RNA isolation and quantitative reverse transcription polymerase chain reaction (qRT-PCR) analysis

Total RNA was extracted from cells or tissues using the TRIzol reagent (Invitrogen, Carlsbad, CA, USA) following the manufacturer’s instructions. Then, 2 μg of each RNA sample was used for cDNA synthesis with the FastKing One Step RT-PCR Kit (Tiangen, Beijing, China). The qRT-PCR was performed in triplicate according to the manufacturer's instructions using the SYBR Green SuperMix system (Bio-Rad Laboratories Inc., Hercules, CA, USA). The gene expression was evaluated for three biological replicates, and the relative changes in gene expression were analyzed by the 2^−ΔΔCT^ method. Glyceraldehyde 3-phosphate dehydrogenase (GAPDH) was used as an internal control. The primer sequences used for qRT-PCR were as follows: human C2orf40: 5′- CCAGCAGTTTCTCTACATGGGC-3′ and 5′-GCAGAGTCTTCATCATAGTGACG-3′; human GAPDH: 5′-GTCTCCTCTGACTTCAACAGCG-3′ and 5′-ACCACCCTGTTGCTGTA GCCAA-3′.

### Western blot analysis

Western blot analysis was performed using standard techniques. Briefly, total proteins from cells or tissues were extracted using radio-immunoprecipitation assay (RIPA) lysis buffer (Millipore, Burlington, MA, USA). Protein concentrations were measured with a Pierce BCA Protein kit (Thermo Fisher Scientific, Waltham, MA, USA). Equal amounts of total lysate were analyzed by sodium dodecyl sulfate–polyacrylamide gel electrophoresis (SDS-PAGE). Proteins were then transferred to polyvinylidene difluoride (PVDF) membranes (IPFL00010; Millipore), blocked with 5% non-fatty milk, and incubated with the appropriate antibodies according to the manufacturer’s instructions. Finally, the blotting was developed using an Enhanced Chemiluminescence (ECL) Detection kit (Merck, Kenilworth, NJ, USA). The band intensity was analyzed by the ImageJ software (National Institutes of Health, Bethesda, MD, USA). The following antibodies were used in the current study: C2orf40 (catalog no. ab224077; Abcam, Cambridge, UK), β-actin(catalog no. ab6276; Abcam), GAPDH (catalog no. ab59164; Abcam), cleaved caspase-3 (catalog no. ab32042; Abcam), caspase-3 (catalog no. ab32351; Abcam), cleaved PARP (catalog no. ab32064; Abcam), PARP (catalog no. ab191217; Abcam), Bax (catalog no. ab32503; Abcam), γ-H2AX (catalog no. ab81299; Abcam), CDK1 (catalog no. ab133327; Abcam), Rb (catalog no. ab181616; Abcam), p-Rb (catalog no. ab184702; Abcam), CCNE1 (catalog no. ab33911; Abcam), CCNB1 (catalog no. ab32053; Abcam), PI3K (catalog no. ab86714; Abcam), AKT (catalog no. ab8805; Abcam), p-AKT (catalog no. ab38449; Abcam), mTOR (catalog no. ab2732; Abcam), p-mTOR (catalog no. ab5536; Abcam), and p-PI3K (catalog no. 17366; Cell Signaling Technology, Inc., Danvers, MA, USA).

### Immunohistochemistry (IHC)

To specifically analyze the expression of the C2orf40, IHC was performed on the paraffin-embedded tumor tissue sections. Briefly, after deparaffinization and rehydration, antigen retrieval was undertaken using citrate solution. The primary antibody (dilution, 1:400; Abcam) was added and incubated overnight at 4 ℃, and a secondary antibody was added and incubated for 30 min in a humid chamber, followed by washing with Tris-buffered saline (TBS) buffer. Slides were then incubated for 30 min with EnVision peroxidase reagent (DAKO, Carpentaria, CA, USA). Finally, the slides were stained with 3, 3-diaminobenzidine (DAB) for 5 min, and Mayer’s hematoxylin solution was used for counterstain. The percentage of positively stained NPC cells in three images was assessed using the ImageJ software as previously described [20].

### Construction of plasmids and lentiviral vectors

Herein, pcDNA-3.1 ( +) vectors (Invitrogen) were used for C2orf40 overexpression. Primers used for amplification of C2orf40 were as follows: C2orf40-F: 5′- CTAGCTAGCCCACCGATGGCTGCCTCCCCCGCGCGGCC-3′ and C2orf40-R: 5′-TTAGTAGTCATCGTAGTTGACGCTGATATCCCG-3′. Besides, AgeI (NEB, Beijing, China) and EcoRI (NEB) enzymes were used to clone the C2orf40 into the pCDNA-3.1( +) vector. For transfection of NPC cells, 3 × 10^5^ NPC cells were seeded into 6-well plates and incubated at 37 ℃ for 24 h. Then, 2000 ng pcDNA-3.1( +) vectors containing C2orf40 or empty vectors were transfected with Lipofectamine^®^ 3000 reagent (Invitrogen). Each experiment was repeated at least three times. For construction of NPC cells stably overexpressing C2orf40, the full-length human C2orf40 gene was subcloned into the lentiviral vector pLV (Add-gene). After that, pLV-C2orf40, psPAX2, and pMD2.G were transiently transfected into HEK-293 T cells. The supernatants that contained viruses were subsequently infected with NPC cells for 48 h. Following infection, the stable clones were selected with 0.5 μg ml^−1^ puromycin (Sigma-Aldrich, St. Louis, MO, USA).

### In vitro chemo-resistance assay

Cell sensitivity to the cisplatin was assessed indirectly by the Cell Counting Kit-8 (CCK-8) and colony formation assays. For CCK-8 assay, transfected NPC cells were seeded into a 96-well plate at a density of 3000 cells/well. After 24 h of incubation, the cells were exposed to cisplatin solution with an appropriate concentration for 3 days according to experimental requirements. Then, cells were incubated with 100 µL of a fresh medium containing 10% CCK-8 reagent (DoJinDo Laboratories, Tokyo, Japan) for 1 h at 37 ℃. The absorbance at a wavelength of 450 nm was detected using an automatic spectrometer (PerkinElmer, Waltham, MA, USA).

For colony formation assay, 600 NPC cells were seeded into a 6-well plate after transfection. After a 24 h-incubation, the cisplatin solution was added to the culture medium in accordance with the experimental conditions. After 14 days, cells were fixed with methanol solution and stained with crystal violet.

### Cell apoptosis

Apoptosis of HONE-1 and SUNE-1 cells induced by cisplatin (1 μg/ml) was determined using an Annexin V-FITC/PI apoptosis detection kit (Beijing 4A Biotech Co., Ltd., Beijing, China) in accordance to the manufacturer’s protocol. The transfected cells were collected and fixed with 4% paraformaldehyde for 1 h at room temperature, and the cells were treated with 0.5% Triton X-100 for 15 min. The TUNEL reaction mixture was placed in a dark environment at 37 °C for 1 h in a humid atmosphere. DAPI was used to stain nuclei simultaneously. The TUNEL-positive NPC cells were analyzed under a fluorescence microscope.

### Bioinformatics analysis

Gene set enrichment analysis (GSEA) was performed using the Molecular Signatures Database (MSigDB; ver. 6.0). Of the 31 NPC patients from GSE12452 and 18 NPC patients from GSE53819, 12 with the highest C2orf40 expression and 12 with the lowest C2orf40 expression were divided into two groups. A *P*-value < 0.05 and a false discovery rate (FDR) < 0.25 were considered significant. Gene Ontology (GO) and Kyoto Encyclopedia of Genes and Genomes (KEGG) pathway enrichment analyses were undertaken using the DAVID (ver. 6.7) website (https://david.ncifcrf.gov).

### Wound healing assay

The migration ability of NPC cells was evaluated by an in vitro wound healing assay. In brief, NPC cells were seeded into 6-well plates at a density of 1 × 10^6^ cells per well in a RIPM-1640 medium supplemented with 10% FBS. After the cells reached a confluence of 80%, the cultured monolayers were mechanically scraped with 200 μl pipette tips and cultured in the RIPM-1640 medium supplemented with 0.5% FBS for 48 h. Images were captured at 0, 12, and 24 h.

### Transwell migration and invasion assays

Transwell migration and invasion assays were performed using transwell chambers (Corning Inc.). Briefly, 5 × 10^4^ HONE-1 or SUNE-1 cells were resuspended on a serum-free RIPM-1640 medium with Matrigel and seeded into the upper chamber of the transwell. Then, the transwell chambers were placed on 24-well plates with a RIPM-1640 medium containing 10% FBS. After incubation at 37 ℃ for 24 h, the cells that invaded the lower chamber of the transwell were fixed with methanol, stained with crystal violet, and photographed under a microscope.

### Immunofluorescence assay

Immunofluorescence assay was carried out to assess the repair ability of cells after radiation injury. Briefly, 5 × 10^4^ HONE-1 or SUNE-1 cells with or without C2orf40 overexpression were seeded, fixed with 4% paraformaldehyde for 10 min, and blocked with 10% bovine serum albumin (BSA) for 15 min. Pooled anti-γ-H2AX antibodies (dilution, 1:100; Abcam) were reacted with the cells for 1 h at 25 °C with gentle mixing. After washing, the bound antibodies were detected by reactivity with secondary antibodies conjugated with fluorescein isothiocyanate for 1 h in the dark. Images were captured using a fluorescence microscope.

### Comet assay

The comet assay was used to assess the oxidative DNA damage in individual cells under radiation exposure. In short, HONE-1 and SUNE-1 cells stably overexpressing C2orf40 or controls were exposed to radiation for the indicated time, and then, the cells were collected (10^6^/ml) and mixed with 0.75% low-melting agarose (Sigma-Aldrich). Afterwards, the cells were spread on a frosted microscopic slide pre-coated with 0.75% normal melting agarose. After solidification of the agarose, cells were lysed with lysis buffer, and the slides were then placed in a gel-electrophoresis apparatus containing electrophoresis buffer (300 mM NaOH and 10 mM Na-EDTA) for 20 min. The electrical field was applied to the same buffer at 4 °C for 20 min to draw the negatively charged DNA towards the anode. After electrophoresis, the slides were rinsed with neutralization buffer and stained with 40 g/mL ETBr (Sigma-Aldrich). The slides were observed under a fluorescence microscope.

### An in vivo mouse model of NPC

Tumor sensitivity to chemotherapy and radiotherapy was evaluated using an in vivo mouse model of NPC. Female nude mice (BALB/c nude mice; age, 6–8-week-old) were used. Besides, 2 × 10^6^ HONE-1 cells were mixed with Matrigel and injected subcutaneously into mice. For cisplatin sensitivity assessment, when the tumor volume reached almost 100 mm^3^, mice were randomly divided into four groups and intraperitoneally injected with phosphate-buffered saline (PBS) or cisplatin (4 mg/kg) every 3 days. Mice were sacrificed on day 30 and tumor volume was calculated using the following equation: (length × width^2^)/2. For the assessment of sensitivity of tumors to radiation therapy, mice were anesthetized via intraperitoneal injection of 150 μl 4% chloral hydrate. When the tumor volume reached almost 100 mm^3^, tumor tissues were exposed to 2-Gy γ rays emitted by the Co-60 source every 2 days from day 1 to day 9, and the growth rate of the tumor was recorded as well. Mice were sacrificed on day 21 and volume and weight of tumors were measured.

### Statistical analysis

The data were imported into SPSS 23.0 (IBM Corp., Armonk, NY, USA) and GraphPad Prism 8.0 (GraphPad Software Inc., San Diego, CA, USA) software for statistical processing, and the results were expressed as mean ± standard deviation (SD) (x̅ ± s). For comparisons, the Wilcoxon signed-rank test, the Pearson chi-square (χ^2^) test, one-way analysis of variance (ANOVA) with Dunnett's test, or the Student’s *t*-test was employed as indicated. *P*-value < 0.05 was considered statistically significant.

## Results

### C2orf40 expression was reduced in NPC cells and associated with a poor prognosis

**Fig. 1:**
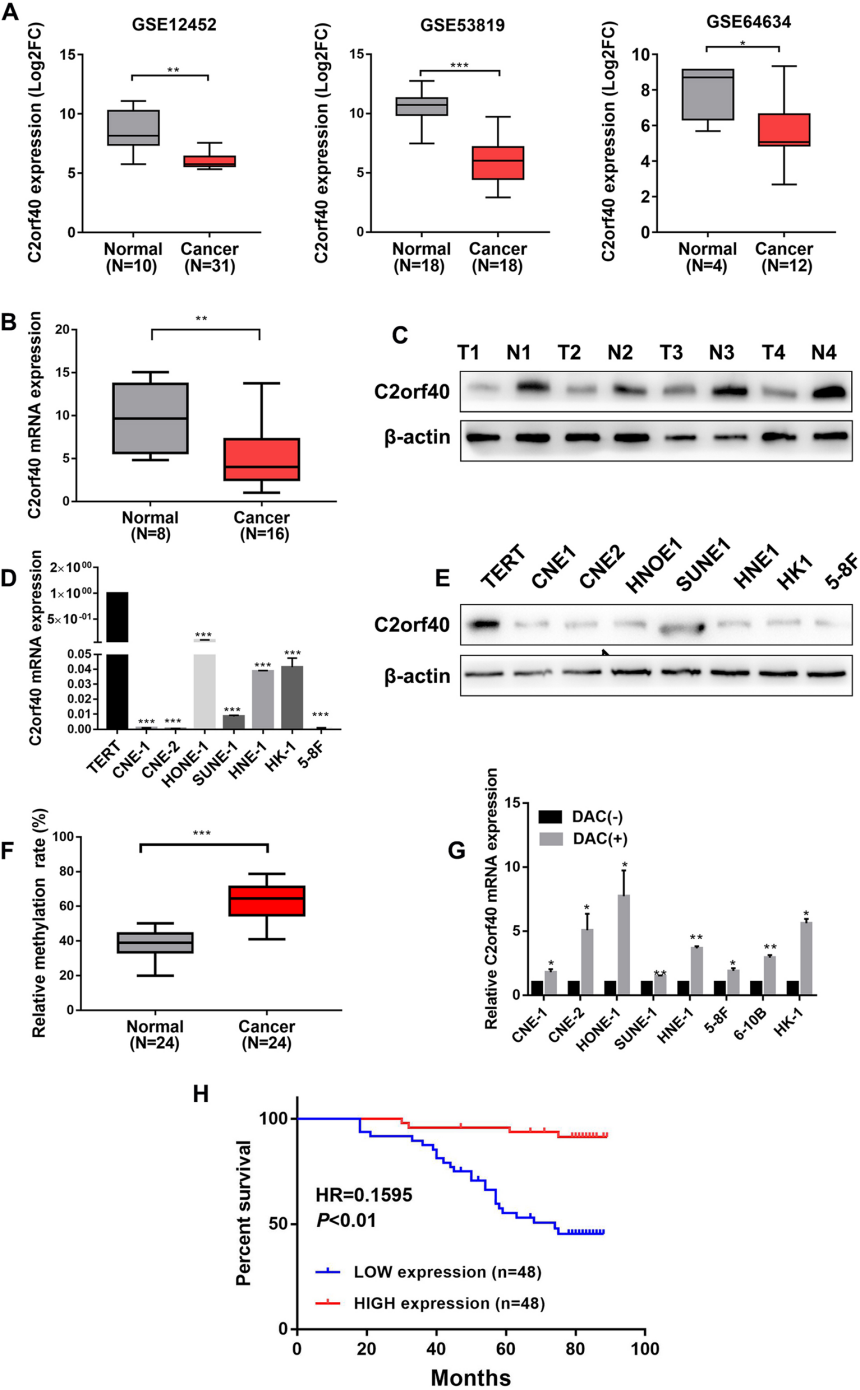
Hypermethylation modified C2orf40 is downregulated in nasopharyngeal carcinoma (NPC) cells and it is associated with a poor prognosis. **A** The expression profile analysis of three GEO datasets (GSE12452, GSE53819, and GSE64634) showed that the C2orf40 expression level in NPC tissues was significantly lower than that in normal nasopharyngeal epithelial tissues. **B** The C2orf40 expression in NPC tissues (*n* = 8) and normal nasopharyngeal epithelial tissues (*n* = 16) was measured by qRT-PCR. **C** Downregulation of C2orf40 expression was confirmed by Western blotting in four matched NPC and adjacent normal tissues. **D‒E** The expression level of C2orf40 was analyzed in one immortalized nasopharyngeal epithelial cell line (TERT) and seven NPC cell lines (CNE-1, CNE-2, HONE-1, SUNE-1, HNE-1, HK-1, and 5-8F) by qRT-PCR (**D**) and Western blotting (**E**). **F** The quantitative methylation levels in 24 paired NPC and normal nasopharyngeal epithelial tissues were detected using pyrosequencing. **G** The NPC cell lines were treated with methyltransferase inhibitor DAC for 48 h, and then, C2orf40 mRNA expression was determined by qRT-PCR. **H** After collecting clinicopathological and follow-up data and conducting immunohistochemistry, the correlations between C2orf40 expression levels and survival prognosis were analyzed in 94 NPC patients. The experiments were performed in triplicate and repeated twice. Data are presented as the mean ± SD. *P < 0.05, **P < 0.01, ***P < 0.001

To identify genes that were differentially expressed in NPC cells, three GEO datasets were integrated into the analysis, including GSE12452 (10 normal samples vs. 31 NPC samples), GSE53819 (18 normal samples vs, 18 NPC samples), and GSE64634 (4 normal samples vs. 12 NPC samples). Of all the differentially expressed genes (DEGs), C2orf40 was identified to be significantly downregulated in NPC tissues compared with that in normal nasopharyngeal epithelial tissues (Fig. 1A, *P* < 0.01). To confirm this result, the C2orf40 mRNA level was determined using the qRT-PCR. The C2orf40 mRNA level in NPC samples (*n* = 16) was markedly lower than that in normal nasopharyngeal epithelial samples (*n* = 8) (Fig. 1B, *P* < 0.01). This result was consistent with that of Western blotting (Fig. 1C). C2orf40 expression in an immortalized nasopharyngeal epithelial cell line (TERT) and seven NPC cell lines (CNE-1, CNE-2, HONE-1, SUNE-1, HNE-1, HK-1, and 5-8F) was also detected using qRT-PCR and Western blotting. As shown in Fig. 1D, E. C2orf40 expression was markedly lower in NPC cell lines, indicating that C2orf40 may play a role in the progression of NPC.

In order to explore why C2orf40 expression decreased in NPC tissues, we first measured the DNA methylation level of C2orf40 gene in 24 NPC tissues and 24 normal nasopharyngeal epithelial tissues using pyrosequencing. The DNA methylation level at the promoter region of C2orf40 in NPC tissues was noticeably higher than that in normal nasopharyngeal epithelial tissues (Fig. 1F, *P* < 0.01). To investigate the direct role of DNA methylation in regulating C2orf40 expression, the NPC cell lines were treated with the DNA methylation inhibitor, decitabine (DAC), and it was found that the C2orf40 mRNA level was significantly elevated in NPC cell lines (Fig. 1G, all *P* < 0.05). Taken together, C2orf40 expression was downregulated in NPC tissues, which is associated with the hypermethylation of its promoter in NPC.

To further determine the role of C2orf40 in the prognosis of NPC patients, IHC was performed. According to the results of IHC, patients with NPC were divided into groups of high C2orf40 expression (*n* = 48) and low C2orf40 expression (*n* = 48). Kaplan–Meier analysis showed that a higher C2orf40 expression predicted a better prognosis (Fig. 1H, hazard ratio (HR) = 0.1595, *P* < 0.01; Table 1). Collectively, C2orf40 could serve as a tumor suppressor gene in NPC.

**Table 1:** Clinical characteristics of NPC patients according to the high and low expression levels of C2orf40

Characteristics	No. of patients	Expression of C2orf40		*P* value
		Low, *n* (%)	High, *n* (%)	
Age				
≤ 45	34	15 (44.1)	19 (55.9)	0.37
> 45	62	32 (51.6)	30 (48.4)	
Sex				
Male	72	37 (51.4)	35 (48.6)	0.409
Female	24	10 (41.7)	14 (58.3)	
T stage				
T1–T2	47	19 (40.4)	28 (59.6)	0.101
T3–T4	49	28 (57.1)	21 (42.9)	
N stage				
N0–N1	27	7 (25.9)	20 (74.1)	< 0.01
N2–N3	69	40 (58.0)	29 (42.0)	
TNM stage				
I–II	13	0 (0)	13 (100.0)	< 0.01
III–V	83	47 (56.6)	36 (43.4)	
Distant metastasis				
No	90	42 (46.7)	48 (53.3)	0.082
Yes	6	5 (83.3)	1 (16.7)	
Death				
Yes	67	22 (32.8)	45 (67.2)	< 0.01
No	29	25 (86.2)	4 (13.8)	

### Overexpression of C2orf40 led to the inhibition of migration of NPC cells

**Fig. 2:**
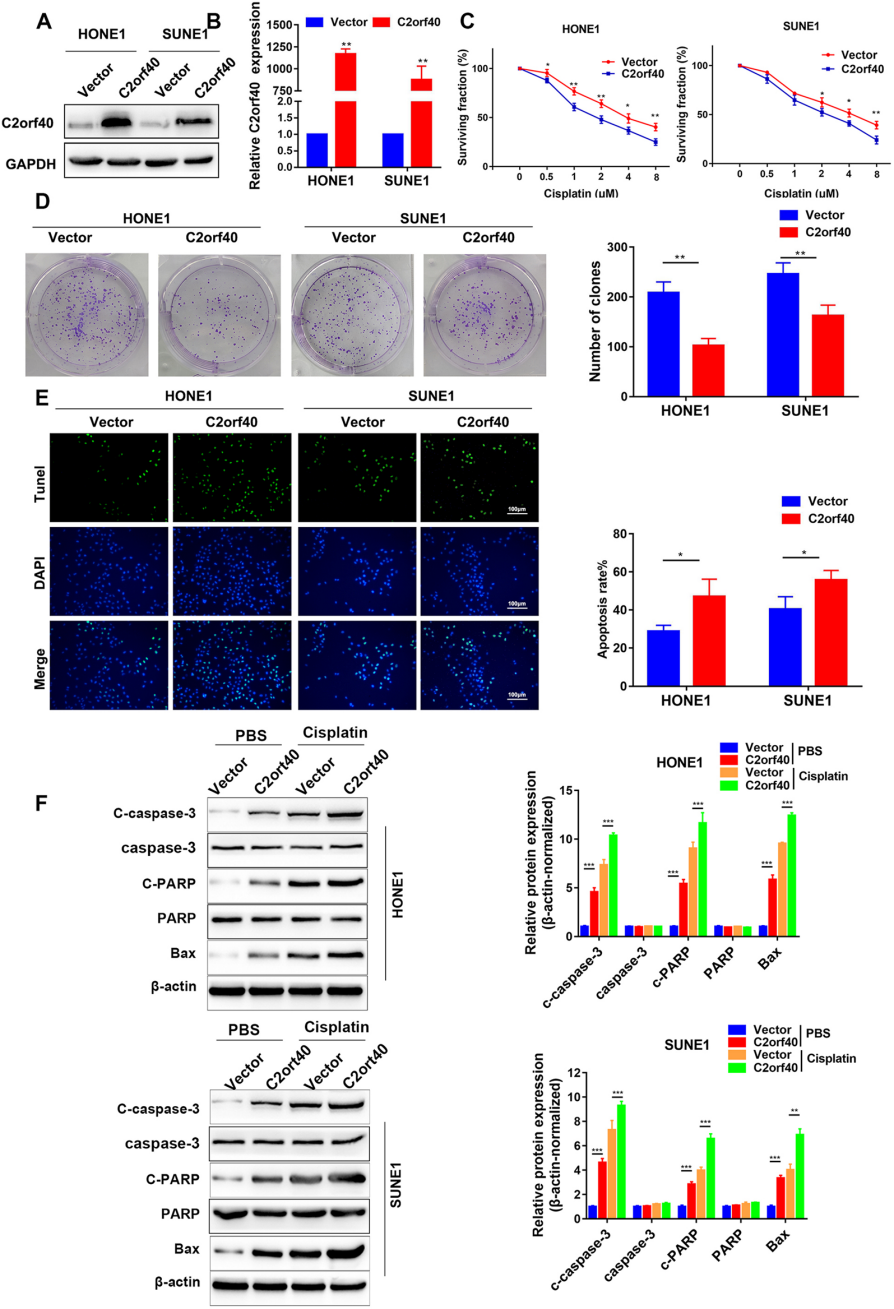
Overexpression of C2orf40 impairs the migration ability of NPC cells in vitro. **A** Gene set enrichment analysis (GSEA) was performed using two GEO datasets (GSE12452 and GSE53819) which compared high C2orf40 expression group and low C2orf40 expression group. The results showed the most significant enrichment of the cell migration-related genes in the high C2orf40 expression group. **B** The migration of HONE-1 and SUNE-1 cells stably overexpressing C2orf40 was assessed by in vitro scratch wound-healing assay. **C** The effects of C2orf40 overexpression on the migration and invasion abilities of SUNE-1 and HONE-1 cells were investigated via transwell migration and invasion assays. The experiments were performed in triplicate and repeated twice. Data are presented as the mean ± SD. *P < 0.05, **P < 0.01, ***P < 0.001

To further clarify the biological functions of C2orf40 in NPC, GESA of GES12452 and GSE53819 datasets was performed. According to the expression level of C2orf40 in NPC samples, these samples were divided into high C2orf40 expression group (top 25%) and low C2orf40 expression group (bottom 25%). GSEA showed that the cell migration-associated genes or signaling pathways were inhibited in high C2orf40 expression group (Fig. 2A, *P* < 0.01). To validate this result, in vitro functional assays were carried out by transfecting NPC cells with C2orf40-overexpression vectors or the empty control vectors. The wound healing assay was undertaken to investigate the role of C2orf40 in the regulation of migration of NPC cells. As shown in Fig. 2B, the migratory rate of HONE-1 and SUNE-1 cells transfected with C2orf40-overexpressing vectors was significantly reduced compared with that of the empty vectors. Additionally, transwell migration and invasion assays revealed that the migration and invasion of NPC cells transfected with C2orf40 overexpression were notably attenuated compared with the control group (Fig. 2C, all *P* < 0.05). Collectively, C2orf40 could inhibit the migration and invasive abilities of NPC cells.

### Overexpression of C2orf40 increased sensitivity of NPC cells to cisplatin

**Fig. 3:**
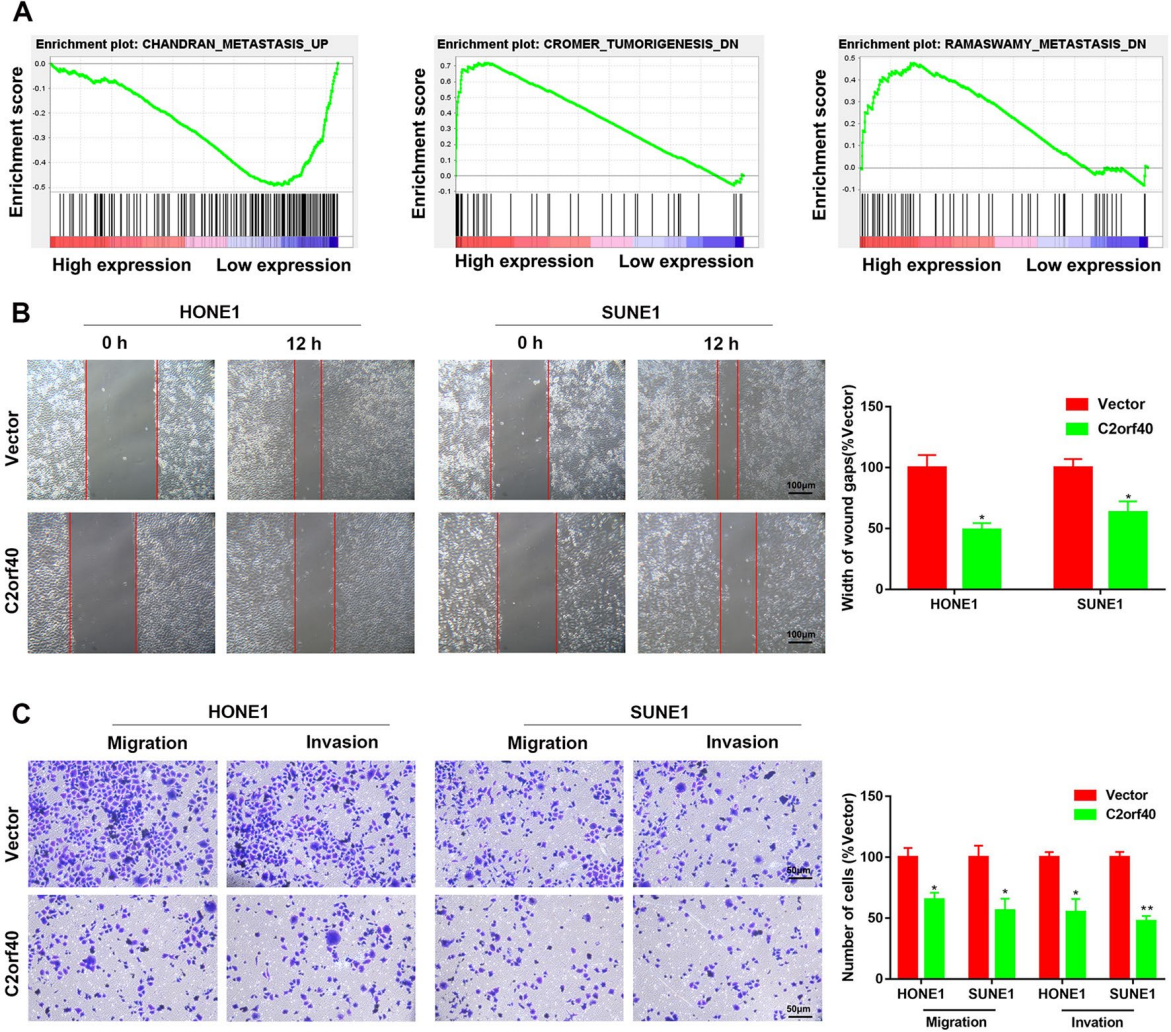
Overexpression of C2orf40 enhances chemo-sensitivity of NPC cells to cisplatin in vitro. **A**‒**B** The endogenous expression level of C2orf40 was determined by Western blotting (**A**) and qRT-PCR (**B**) after stably overexpressing C2orf40 in SUNE-1 and HONE-1 cells. **C** SUNE-1 and HONE-1 cells were pretreated with cisplatin for the indicated concentrations, and then, they were subjected to CCK-8 assay. **D–F** SUNE-1 and HONE-1 cells were exposed to 1 µg/ml cisplatin for 48 h. **D** The sensitivity of NPC cells to cisplatin was evaluated using colony formation assay. **E** The apoptosis level of NPC cells was assessed using TUNEL staining. The results were shown as a percentage of TUNEL + cells versus total cells. **F** Western blot analysis of the expression levels of apoptosis-related genes (C-caspase-3, C-PARP, and Bax) in NPC cells. The β-actin protein was used as loading control. The experiments were performed in triplicate and repeated twice. Data are presented as the mean ± SD. *P < 0.05, **P < 0.01, ***P < 0.001

To gain a better understanding of the biological function of C2orf40 in NPC, HONE-1 and SUNE-1 cells stably overexpressing C2orf40 were tested. The mRNA and protein levels of C2orf40 were then investigated by Western blotting (Fig. 3A) and qRT-PCR (Fig. 3B, *P* < 0.01). The effects of C2orf40 on the sensitivity of cells to cisplatin were also evaluated. HONE-1 and SUNE-1 cell lines were evaluated by the CCK-8 assay with various concentrations of cisplatin (0‒8 μM). The results showed that NPC cells stably overexpressing C2orf40 were more sensitive to the cisplatin treatment (Fig. 3C). These results were consistent with those achieved by colony formation assay, which showed that the colonies of NPC cells stably overexpressing C2orf40 were more sensitive to treatment with cisplatin (Fig. 3D, *P* < 0.01). Moreover, TUNEL assay was used to determine the cisplatin‐induced apoptosis of NPC cells. It was found that C2orf40 overexpression increased the number of TUNEL-positive NPC cells, enhancing cisplatin-induced apoptosis (Fig. 3E, *P* < 0.05). In order to further verify the effects of C2orf40 on cisplatin-induced cell apoptosis, we detected the expression levels of apoptosis-associated proteins using Western blotting. HONE-1 and SUNE-1 cells were exposed to cisplatin (1 μg/ml) or PBS (mock treatment) for 48 h. Western blot analysis indicated that C2orf40 overexpression remarkably increased the expression levels of apoptosis-associated proteins, including C-caspase-3, C-PARP, and Bax. More importantly, cisplatin treatment could significantly upregulate the expression levels of these proteins (Fig. 3F, *P* < 0.01). Taken together, C2orf40 could enhance the chemo-sensitivity of NPC cells to cisplatin by promoting the cell apoptosis.

### Overexpression of C2orf40 increased the sensitivity of NPC cells to radiotherapy

**Fig. 4:**
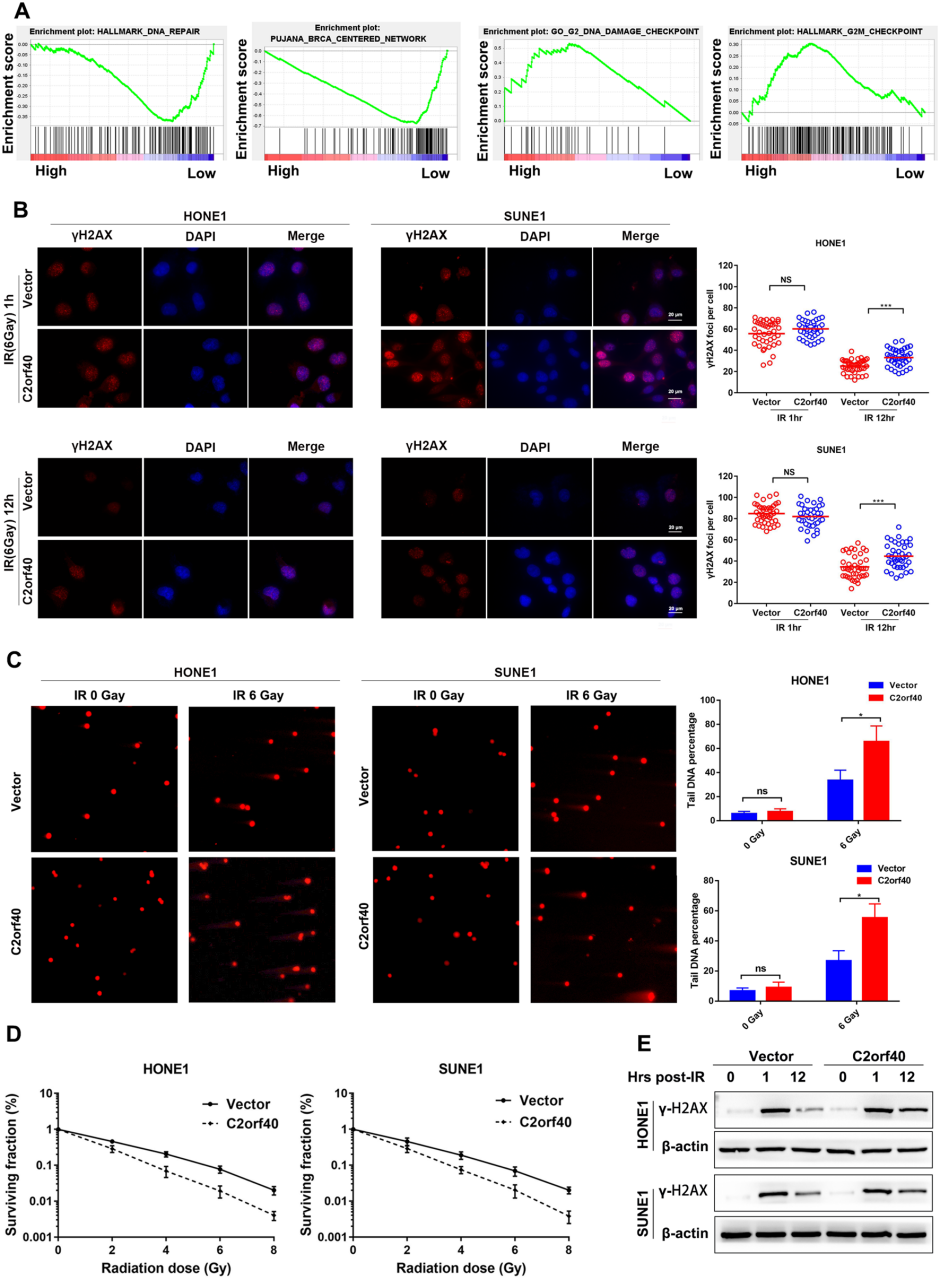
C2orf40 overexpression promotes the sensitivity of NPC cells to radiotherapy in vitro. **A** GSEA was performed using two GEO datasets (GSE12452 and GSE53819) as mentioned previously. The results demonstrated the enrichment of the gene signatures associated with DNA repair. **B‒C** HONE-1 and SUNE-1 cells stably overexpressing C2orf40 or controls were submitted to X-ray irradiation (IR). **B** Concurrent staining for the DNA damage marker γH2AX and endogenous control DAPI revealed that γH2AX expression, as measured by immunofluorescence, was significantly higher after 24 h of 6-Gy IR in NPC cells with C2orf40 overexpression. **C** Endogenous DNA damage and repair were assessed by the comet assay, indicating that overexpression of C2orf40 impaired the ability of NPC cells to repair DNA damage. **D** HONE-1 and SUNE-1 cells stably overexpressing C2orf40 or controls were exposed to a range of X-ray doses: 0, 2, 4, 6, and 8 Gy. Then, the clonogenic cell survival was measured to evaluate the radiation sensitivity of NPC cells. **E** Western blotting showed the γH2AX protein levels in HONE-1 and SUNE-1 cells after exposure to 6-Gy IR for 0, 1, and 12 h, with β-actin as an internal reference. The experiments were performed in triplicate and repeated twice. Data are presented as the mean ± SD. *P < 0.05, **P < 0.01, ***P < 0.001

GESA of GES12452 and GSE53819 datasets also indicated that C2orf40 expression was correlated with the activation of DNA repair pathways (Fig. 4A, all *P* < 0.01). As disappearance of lesions induced by DNA damage also reflects the effectiveness of DNA repair pathways [21], we analyzed radiation-induced γH2AX foci using immunofluorescence (IF) assay. γH2AX is an H2A variant that could be phosphorylated by ATM and other PIKK on the Ser139 of the DNA damage site. This process occurs a few minutes after radiation, reaches a peak after 20 min, and disappears within 8 h after radiation [22]. As expected, at half an hour after irradiation, there was no significant difference in the amount of intracellular γH2AX between the C2orf40 overexpression group and the control group, while a higher level of γH2AX was found in NPC cells with C2orf40 overexpression at 12 h after irradiation (Fig. 4B, *P* < 0.01). Meanwhile, the results of comet assay revealed that, C2orf40 overexpression increased the length of comet tails under irradiation treatment (6 Gy), indicating that C2orf40 could enhance DNA damage (Fig. 4C, *P* < 0.01). Based on the above-mentioned results, we conducted a clonogenic cell survival assay to assess the influence of C2orf40 on the radio-sensitivity of NPC cells. As shown in Fig. 4D, no significant difference was detected in colonies between NPC cells with or without C2orf40 overexpression before radiotherapy, while noticeable differences were identified in colonies at doses of 2 to 8 Gy after radiotherapy. The Western blotting simultaneously revealed that γH2AX expression was more elevated in NPC cells with C2orf40 overexpression (Fig. 4E). Overall, the results demonstrated that the aberrant C2orf40 expression could influence the sensitivity of NPC cells to radiation.

### Overexpression of C2ORF40 induced cell cycle arrest at the G2/M phase

**Fig. 5:**
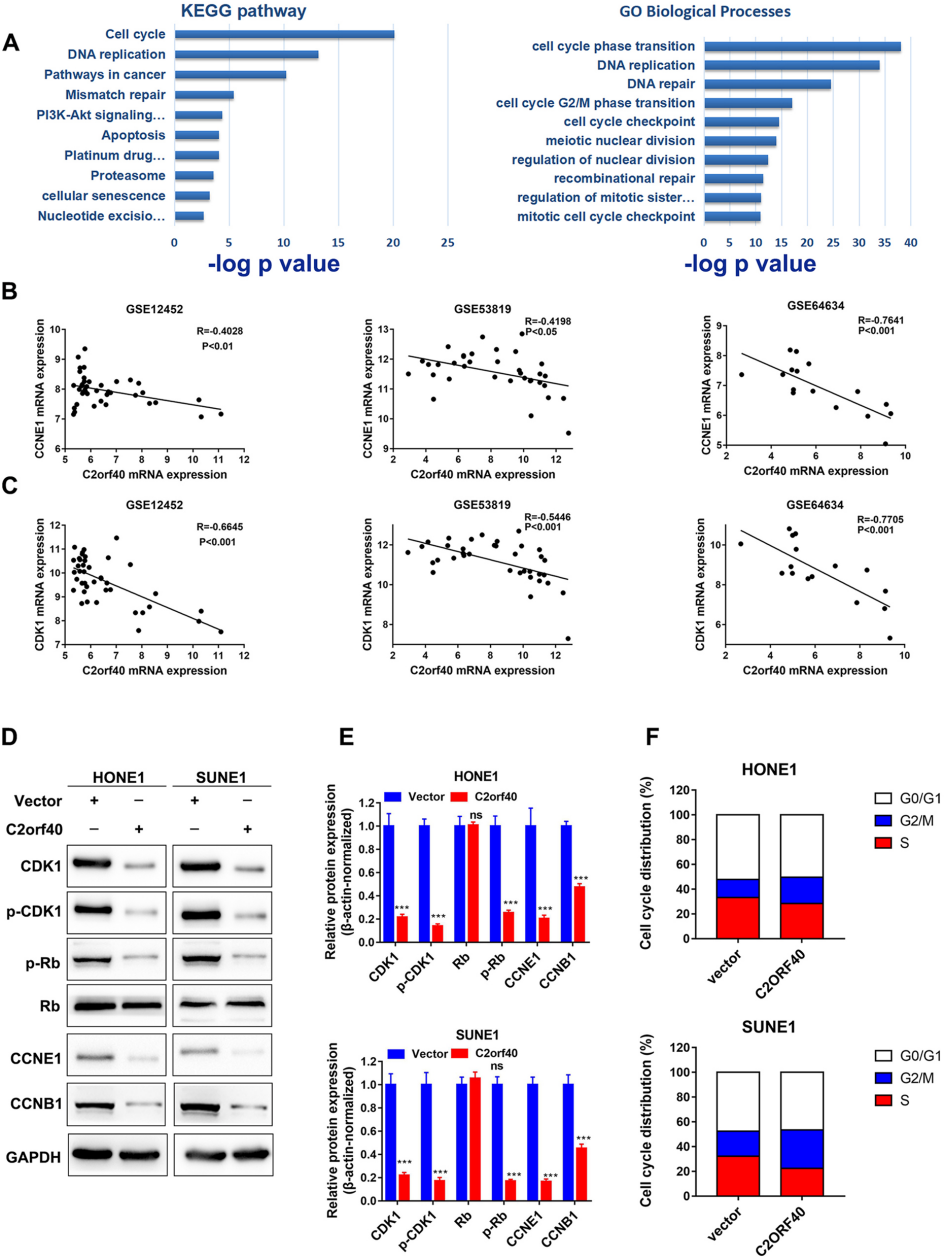
C2orf40 overexpression in NPC cells could induce the cell cycle arrest at G2/M phase. **A** GO and KEGG pathway enrichment analyses of 544 genes that were negatively correlated with C2orf40 expression (|R|> 0.3, *P* < 0.05) using Metascape. Data were imported from the three GEO datasets (GSE12452, GSE53819, and GSE64634). **B‒C** A strong inverse correlation of C2orf40 expression levels with the expression levels of both CCNE1 (**B**) and CDK1 (**C**) was noted in the three GEO datasets. **D‒E** The representative images (**D**) and statistical analysis (**E**) of Western blotting of the expression levels of cell cycle-related genes (CDK1, p-CDK1, p-Rb, Rb, CCNE1 and CCNB1) in HONE-1 and SUNE-1 cells. GAPDH was used as an internal reference. **F** Cell cycle analyses carried out by flow cytometry of HONE-1 and SUNE-1 cells showed a cell cycle arrest at G2/M phase after transiently transfecting C2orf40 plasmids. The experiments were performed in triplicate and repeated twice. Data are presented as the mean ± SD. *P < 0.05, **P < 0.01, ***P < 0.001

In order to explore the potential mechanisms underlying how C2orf40 could mediate the migration ability, chemo-sensitivity, and radio-sensitivity of NPC cells, we first searched for genes which were inversely correlated with C2orf40 based on the above-mentioned GEO datasets (GSE12452, GSE53819, and GSE12452). A total of 544 genes were screened out by determining the expression levels of genes that were inversely correlated with the expression level of C2orf40 (|R|> 0.3, *P* < 0.05). The GO and KEGG pathway enrichment analyses were subsequently conducted. It was found that the expression levels of 544 genes were negatively correlated with the expression level of C2orf40, and were significantly associated with cell cycle regulation, platinum resistance, DNA repair, and PI3K signaling pathway (Fig. 5A, *P* < 0.01). Among them, the expression levels of cell cycle-related proteins, CCNE1 and CDK1, were negatively correlated with the expression level of C2orf40 in the three GEO datasets (Fig. 5B, C, *P* < 0.05), suggesting that C2orf40 was involved in the regulation of cell cycle in NPC. For verification, Western blotting was employed to indicate whether C2orf40 could influence the expression levels of cell cycle-associated proteins, including CDK1, p-CKD1, p-Rb, CCNE1 and CCNB1 [23–25]. After overexpression of C2orf40, the expression levels of CDK1, CCNE1 and CCNB1 in NPC cells were markedly downregulated. At the same time, the phosphorylation levels of CDK1 and Rb proteins were inhibited by C2orf40 overexpression (Fig. 5D, E). Furthermore, flow cytometry analysis showed that overexpression of C2orf40 induced cell cycle arrest at the G2/M phase in HONE-1 and SUNE-1 cells (Fig. 5F). Collectively, these C2orf40 induced cell cycle arrest at the G2/M phase.

### C2orf40 inhibited homologous recombination repair and PI3K/AKT/mTOR signal pathway

**Fig. 6:**
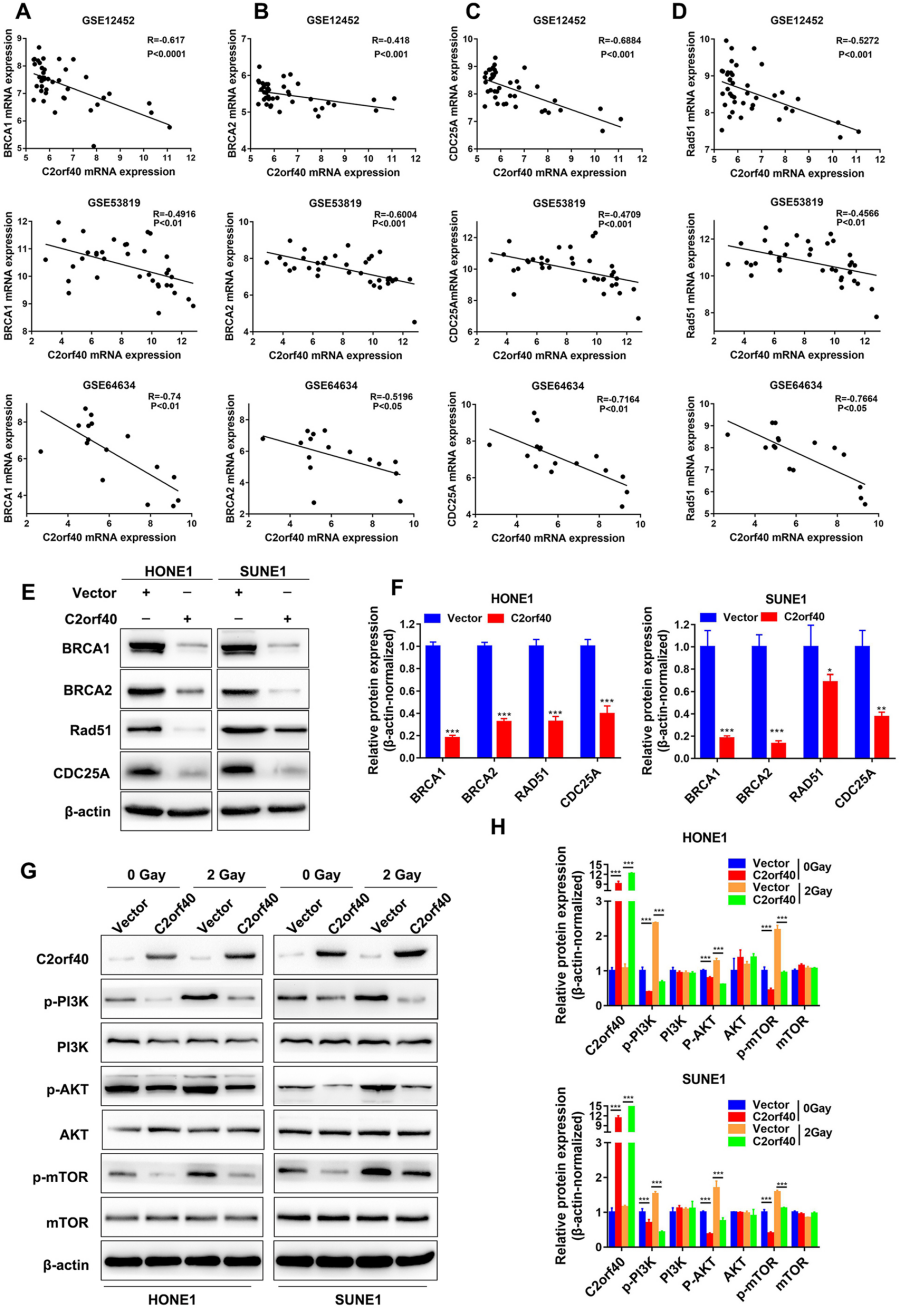
C2orf40 could down-regulate the expression levels of homologous recombination-associated proteins and inhibit the activation of PI3K/Akt/mTOR signaling pathway. **A**‒**D** Correlation analysis revealed that the C2orf40 expression level was negatively correlated with the expression levels of BRCA1 (**A**), BRCA2 (**B**), CDC25A (**C**), and RAD51 (**D**). The data were obtained from three GEO datasets (GSE12452, GSE53819, and GSE64634). **E‒F** The representative images (**E**) and statistical analysis (**F**) of Western blotting of the expression levels of homologous recombination-associated proteins (BRCA1, BRCA2, RAD51, and CDC25A) in HONE-1 and SUNE-1 cells transiently transfected with C2orf40-overexpressed plasmids. β-actin was used as an internal reference. **G‒H** The representative images. (**G**) and statistical analysis (**H**) of the activation of the PI3K/Akt/mTOR signaling pathway. HONE-1 and SUNE-1 cells were transiently transfected with C2orf40 overexpression vectors or empty vectors, and exposed to 0 or 2 Gy radiation. The experiments were performed in triplicate and repeated twice. Data are presented as the mean ± SD. *P < 0.05, **P < 0.01, ***P < 0.001

Based on the results of gene co-expression analysis, we identified a negative correlation between the expression levels of C2orf40 and genes that were related to homologous recombination repair (HRR), including BRCA1 (Fig. 6A, *P *< 0.01), BRCA2 (Fig. 6B,* P* < 0.05), CDC25A (Fig. 6C, *P* < 0.01), and RAD51 (Fig. 6D, *P* < 0.05). These findings demonstrated that C2orf40 could promote the sensitivity of NPC cells to radiotherapy and chemotherapy by inhibiting the expression levels of HRR-related proteins. Consistently, Western blotting revealed that overexpression of C2orf40 in HONE-1 and SUNE-1 cells down-regulated the expression levels of BRCA1, BRCA2, RAD51, and CDC25A (Fig. 6E, F, *P* < 0.05). Additionally, the results of bioinformatics analysis indicated that C2orf40 could contribute to the activation of the PI3K/AKT/mTOR signaling pathway (Fig. 5A). For verification, the NPC cells were exposed to 0 or 2 Gy irradiation and the activation of PI3K/AKT/mTOR signaling pathway was evaluated via Western blotting. In the absence of radiation, overexpression of C2orf40 inhibited the phosphorylation levels of PI3K, AKT, and mTOR. When the cells were irradiated at 2 Gy, these inhibitory effects still existed (Fig. 6G, H*, P* < 0.01). Taken together, C2orf40, as a tumor suppressor gene, could inhibit the migration ability of NPC cells and enhance the sensitivity of NPC cells to chemotherapy and radiotherapy through inhibiting HRR and the PI3K/Akt/mTOR signaling pathway.

### Overexpression of C2orf40 increased the sensitivity of NPC cells to chemotherapy and radiation therapy in vivo

**Fig. 7:**
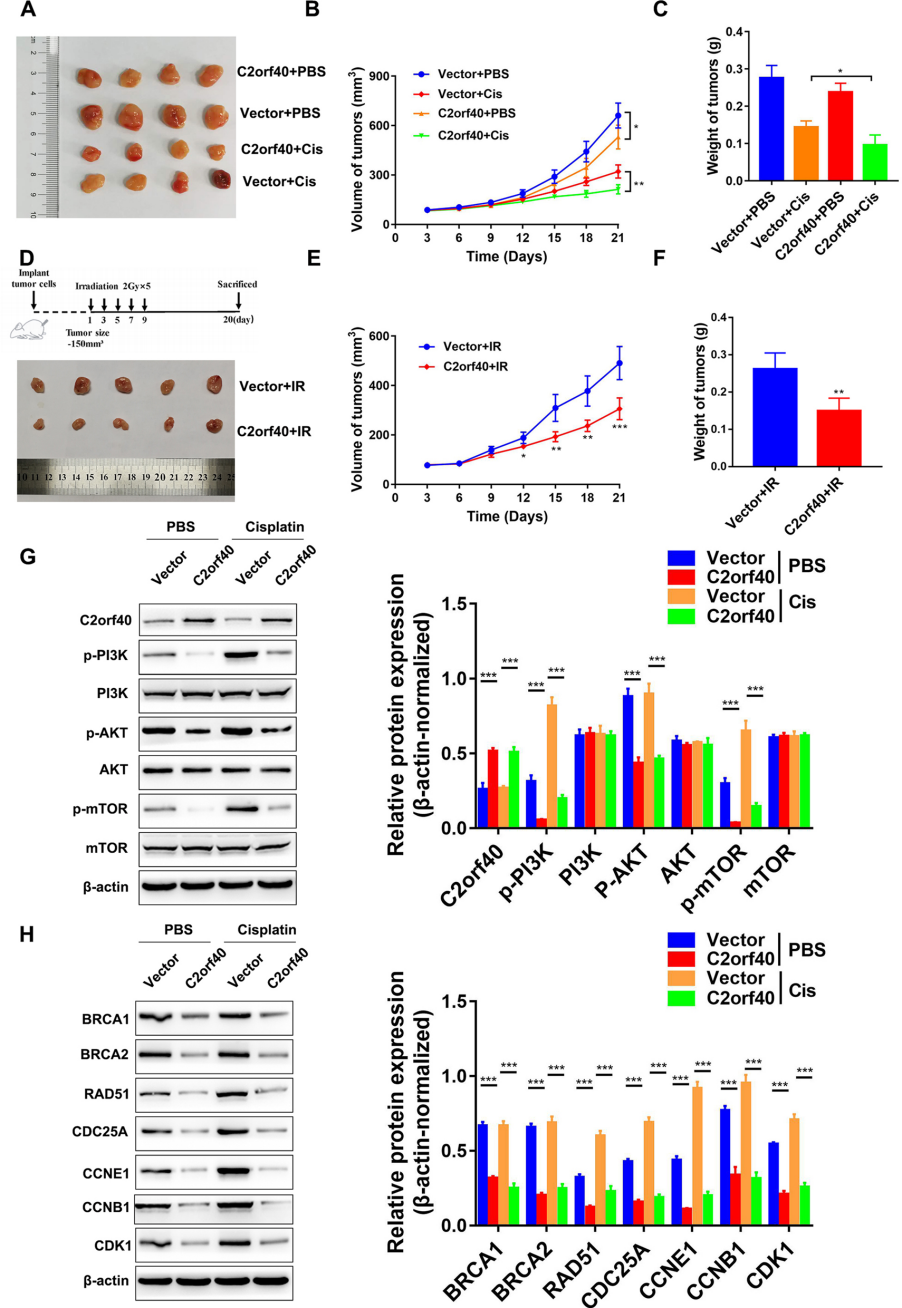
Overexpression of C2orf40 results in increased chemo-sensitivity and radio-sensitivity of NPC cells in vivo. **A**‒**C** An in vivo mouse xenograft tumor model was established by subcutaneous injection of HONE-1 cells into nude mice treated with PBS or cisplatin (4 mg/kg, once every 3 days). **A** Representative images of xenograft tumors resected from the xenograft model mice. The tumor volume (**B**) and tumor weight (**C**) were measured in xenograft tumors from nude mice. **D‒F** HONE-1 cells were subcutaneously injected into nude mice to establish the transplanted tumor model. When the tumor volume reached 150 ± 25 mm^3^, a local dose of 2.0 Gy was given 5 times. **D** Xenograft tumors were resected from mice after 21 days of injection of the cells. **E** Time–growth curves of tumor xenografts. **F** Weight of xenograft tumors harvested from mice. Data are presented as the mean ± SD. **G** Western blotting of the activation of PI3K/Akt/mTOR signaling pathway in xenografts. **H** Western blotting was used to detect the expression levels of cell cycle-related genes in xenografts. *P < 0.05, **P < 0.01, ***P < 0.001

In order to evaluate the influences of C2orf40 on chemo-resistance and radio-resistance of NPC cells, we conducted in vivo experiments. BALB/c nude mice were subcutaneously injected with HONE-1 cells. Then, mice received 4 mg/kg cisplatin intraperitoneally two or three times per week. The tumor growth was measured every 3 days. Finally, the tumor growth rate and tumor weight were used to evaluate sensitivity of tumor to cisplatin chemotherapy (Fig. 7A). As excepted, significant reductions in tumor growth rate and tumor weight were observed in the cisplatin treatment group compared with those in PBS-treated mice. After cisplatin treatment, overexpression of C2orf40 resulted in noticeable retardations in tumor growth and tumor weight compared with the control group (empty vectors) (Fig. 7B, C). These findings indicated that C2orf40 was a tumor suppressor gene, enhancing the chemo-sensitivity of NPC cells.

To further investigate the significance of C2orf40 in tumor radio-sensitivity, HONE-1 cells were injected into nude mice to establish a subcutaneous tumor model. The tumors were exposed to 2 Gy radiation (five times) and the tumor growth rate was recorded (Fig. 7D). The results showed that compared with the control group, the overexpression of C2orf40 decreased the tumor growth rate after radiation (Fig. 7E). In addition, overexpression of C2orf40 reduced the tumor weight after radiation (Fig. 7E, *P* < 0.01). Besides, C2orf40 inhibited the phosphorylation levels of PI3K, AKT, mTOR, and cell cycle-related proteins in tumor cells (Fig. 7G, H). Taken together, C2orf40 played important roles in tumor growth after radiotherapy.

## Discussion

NPC is a common type of cancer in Africa and Southeast Asia, highly associated with genetic factors, dietary effects, and viral infections [1, 26]. At present, intensity-modulated radiation therapy (IMRT) combined with platinum-based chemotherapy is still the main treatment choice for patients with NPC [27–29]. However, several factors, including recurrence, distant metastasis, and radiation resistance, may reduce the efficacy of radiotherapy and chemotherapy in the treatment of NPC [5, 30]. Therefore, a new treatment regimen aimed at improving the sensitivity of radiotherapy and chemotherapy for NPC is expected to improve the overall survival rate and quality of life of patients with NPC. We attempted to investigate the C2orf40 gene because when we reanalyzed the publicly accessible NPC microarray dataset previously stored in the GEO database, it was significantly downregulated in all the three datasets. More importantly, the biological function and mechanism of C2orf40 in NPC have not been reported.

The C2orf40 gene encodes a secretory protein, which is hydrolyzed to produce soluble peptides, and considered to be necessary for C2orf40 to exert its cell type-specific biological activity [31]. In recent years, a number of scholars have found that C2orf40 is a tumor suppressor gene, which has a variety of functions in the processes of cell proliferation, migration, and invasion. In breast cancer, C2orf40 is mainly silenced by hypermethylation of the promoter. The mRNA level of C2orf40 was significantly correlated with disease-free survival and distant metastasis-free survival. C2orf40 inhibits the proliferation, migration, and invasion of breast cancer cells by down-regulating the expression levels of mitotic genes [32]. In esophageal cancer, soluble C2orf40 has a dose-dependent inhibitory effect on the growth of esophageal cancer cells in vivo. Additionally, C2orf40 inhibits the growth of tumor cells by reducing telomerase activity and it is therefore expected to be a potential biotherapeutic drug for esophageal cancer. In the present study, the results not only showed that the expression level of C2orf40 in NPC cells was down-regulated, but also revealed that the expression level of C2orf40 was highly correlated with the prognosis of NPC patients. The downregulation of C2orf40 gene in NPC cells could be partially due to its hypermethylated promoter. In addition, as a potential tumor suppressor gene, C2orf40 inhibits the expression levels of cell cycle-related proteins (CCNE1 and CDK1), and blocks the cell cycle in G2/M phase. Furthermore, C2orf40 inhibits the resistance and migration of NPC cells to radiotherapy and chemotherapy by downregulating the expression levels of HRR-related proteins and activation of PI3K/Akt/mTOR signaling pathway.

Epigenetics refers to the change of genetic gene expression without changing DNA sequence. It includes DNA methylation, histone modification, nucleosome remodeling, and RNA-mediated targeting [33, 34]. Among them, DNA methylation is the most common epigenetic change [35]. Methylation of CpG island in the promoter or the first exon inhibits gene transcription and leads to inactivation of gene expression [36, 37]. With the development of sequencing and microarray technologies, we can easily screen the expression and DNA methylation levels of thousands of genes in the human genome simultaneously. DNA methylation is closely associated with the regulation of gene expression, and the high expression levels of oncogenes and low expression levels of tumor suppressor genes are the key factors of tumorigenesis [38]. Perturbation of DNA methylation is common in various types of cancer, and it has become an important mechanism of tumorigenesis. Aberrant DNA methylation may influence the functions of key genes, especially tumor suppressor genes, thereby participating in the carcinogenesis of NPC. To date, several studies have reported some hypermethylated genes in NPC, and described the overall outline of the interaction network of these aberrantly methylated genes, providing a valuable reference for the study on occurrence and development of NPC at the molecular level [39–41]. Particularly, the promoter hypermethylation of SHISA3 contributes to the downregulation of this gene in NPC. SHISA3 suppresses invasion and metastasis of NPC cells by impeding the TRIM21-mediated ubiquitination and degradation of SGSM1 [42]. HOPX gene is the most prominent hypermethylated gene in NPC, and restoring the expression level of HOPX inhibits the metastasis of NPC cells and enhances their sensitivity to chemotherapy [43]. Moreover, numerous scholars demonstrated that the down-regulation of C2orf40 expression in tumor cell lines and tissues is mainly attributed to the hypermethylation of its promoter [31, 44–46]. Consistently, in the present study, through pyrosequencing, it was found that the promoter region of C2orf40 was hypermethylated, which indicated its down-regulated expression level in NPC cells.

In conclusion, This study clarified the biological functions and mechanisms of C2orf40, as a tumor suppressor gene, in NPC, and provided a potential molecular target for improving the sensitivity of NPC cells to radiotherapy and chemotherapy. it may be a promising treatment for NPC to restore the expression level of C2orf40 in NPC cells by epigenetic therapy or the application of recombinant C2orf40-derived peptides.

## Data Availability

The datasets used and/or analysed during the current study are available from the corresponding author on reasonable request.

## Abbreviations

NPC: Nasopharyngeal carcinoma
ECRG4: Esophageal cancer-related gene-4 protein
ESCC: Esophageal squamous cell carcinoma
SDS-PAGE: Sodium dodecyl sulfate–polyacrylamide gel electrophoresis
ECL: Enhanced chemiluminescence
IHC: Immunohistochemistry
CDS: Coding sequence
CCK-8: Cell counting kit-8
GSEA: Gene set enrichment analysis
MSigDB: Molecular signatures database
FDR: False discovery rate
GO: Gene ontology
KEGG: Kyoto encyclopedia of genes and genomes
LMA: Low-melting agarose
qRT-PCR: Quantitative reverse transcription-polymerase chain reaction
IF: Immunofluorescence
HRR: Homologous recombination repair
IMRT: Intensity-modulated radiation therapy
